# Supplementary material for: Molecular Characterization of Non-Neurogenic and Neurogenic Lower Urinary Tract Dysfunction (LUTD) in SCI-Induced and Partial Bladder Outlet Obstruction Mouse Models
Source: Int J Mol Sci. 2023 Jan 26;24(3):2451. doi: 10.3390/ijms24032451 (PMC9916488; doi:10.3390/ijms24032451)
Supplement: Supplementary file 1 [file ijms-24-02451-s001.zip › Supplementary_figures_Monastyrskaya.pdf]

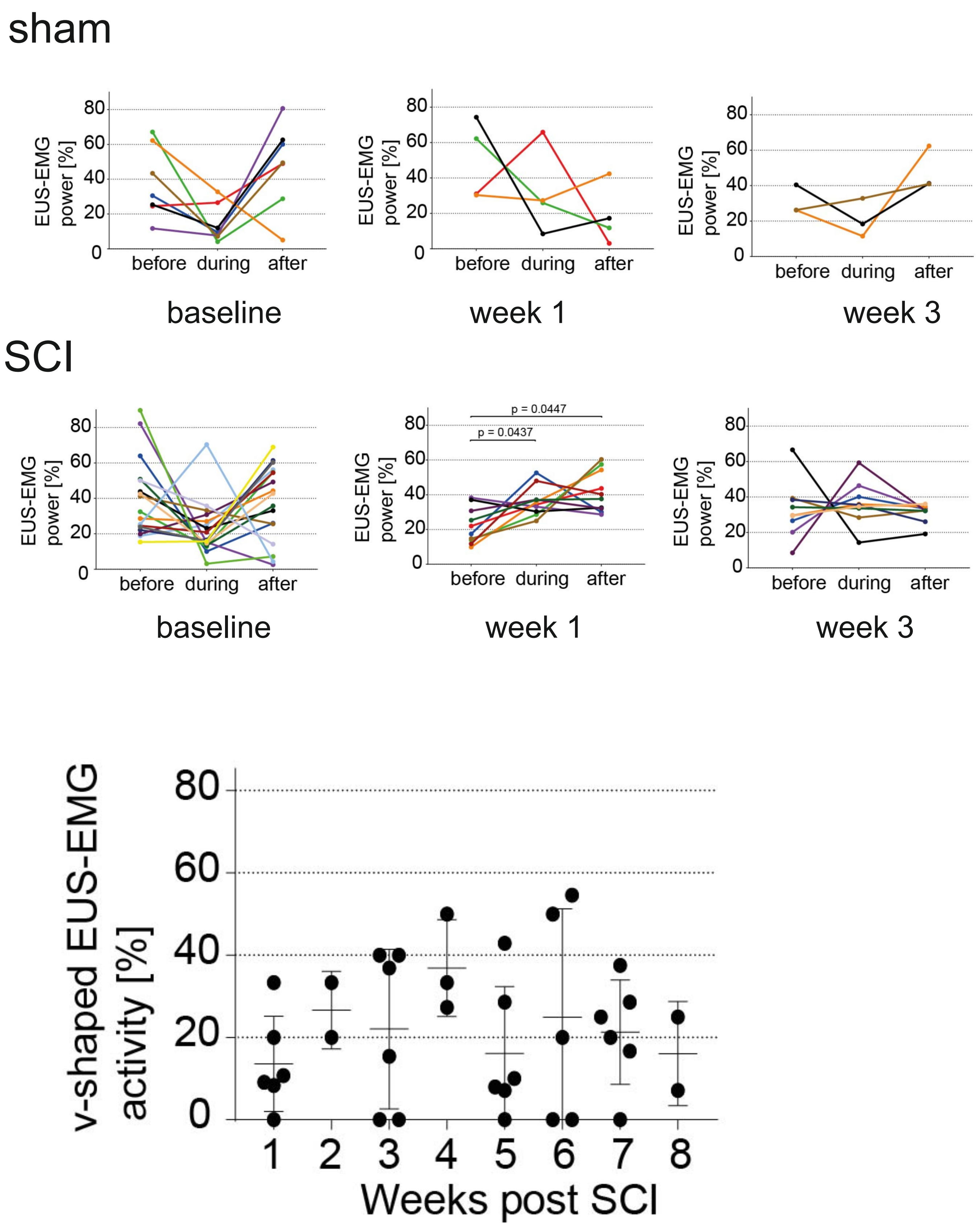
sham
EUS-EMG power [%]
80
60
40
20
0
before
during
after
baseline
week 1
week 3
SCI
p = 0.0447
p = 0.0437
v-shaped EUS-EMG activity [%]
1
2
3
4
5
6
7
8
Weeks post SCI

Fig. 1S

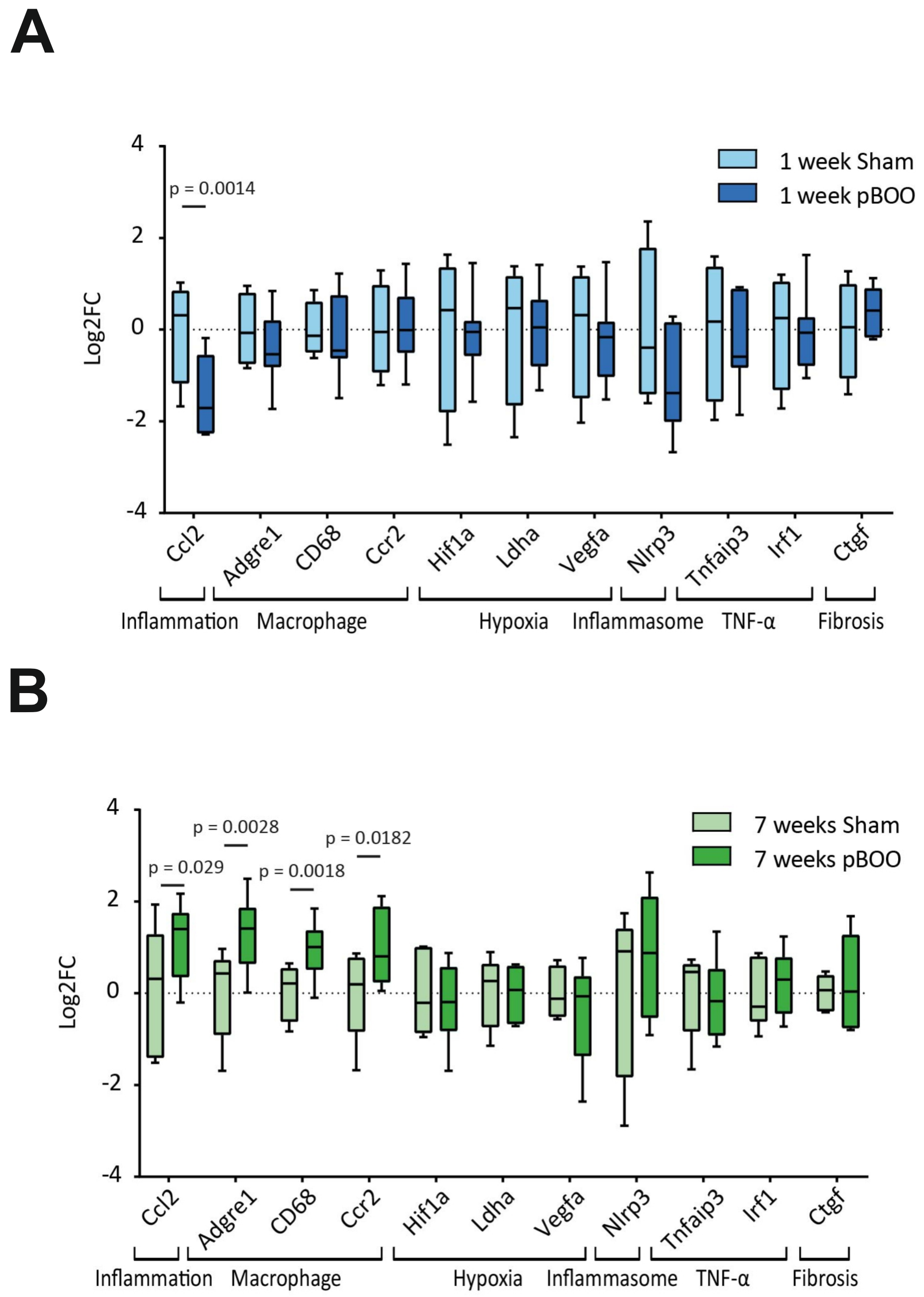
A
4
2
0
-2
-4
Log2FC
p = 0.0014
1 week Sham
1 week pBOO
Ccl2
Adgre1
CD68
Ccr2
Hif1a
Ldha
Vegfa
Nlrp3
Tnfaip3
Irf1
Ctgf
Inflammation
Macrophage
Hypoxia
Inflammasome
TNF-α
Fibrosis
B
4
2
0
-2
-4
Log2FC
p = 0.029
p = 0.0028
p = 0.0018
p = 0.0182
7 weeks Sham
7 weeks pBOO
Ccl2
Adgre1
CD68
Ccr2
Hif1a
Ldha
Vegfa
Nlrp3
Tnfaip3
Irf1
Ctgf
Inflammation
Macrophage
Hypoxia
Inflammasome
TNF-α
Fibrosis

Fig. 2S

Reactome

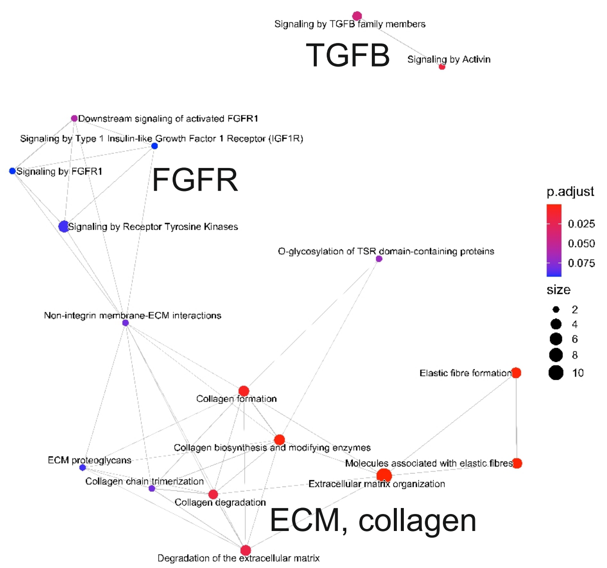

KEGG

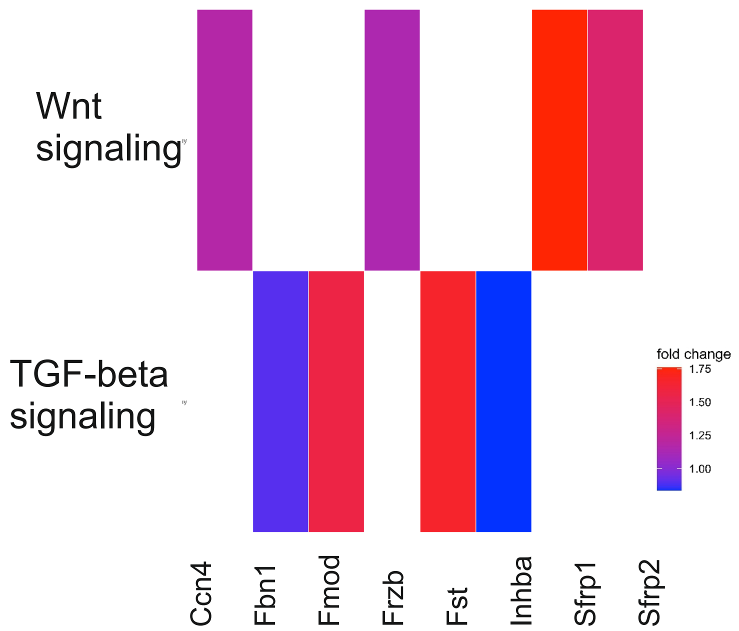

GO CC

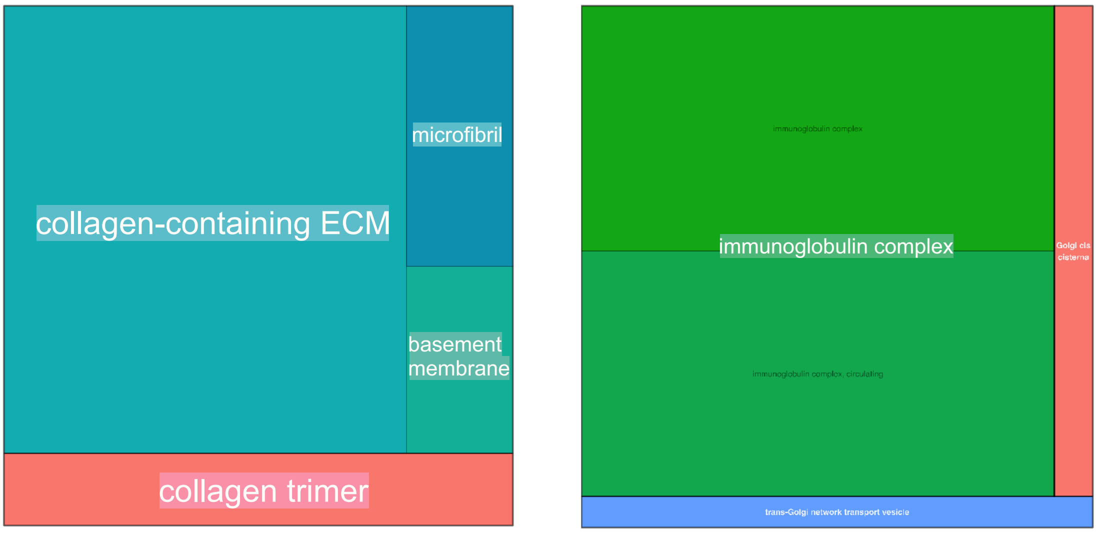

46 common DEGs, same regulation

13 common DEGs, up in pBOO, down in SCI

Fig. 3S
